# BJORL - Its History until 1996

**DOI:** 10.5935/1808-8694.20130024

**Published:** 2015-11-02

**Authors:** Ricardo Ferreira Bento

**Affiliations:** Full Professor of Otorhinolaryngology - Medical School of the University of São Paulo (USP)

To write about the beginnings of the Brazilian Journal of Otorhinolaryngology, back in 1933 through 1990, feels like recounting the very history of our specialty in Brazil. In the capacity of Editor, between 1989 and 1996, I strengthened my ties with the publication and became interested in all it represented to Otorhinolaryngology. When I was the Editor, my first job was to prevent the journal from not being published, so that it could grow and achieve relevance, together with national and international exposure. Some years before, in 1989, when I took on the challenge, the journal had three papers, usually review papers or case reports, published in each one of the three annual issues. There were no advertisements and the medical journal - which is among the five oldest papers in Brazil - was all paid for by the late Brazilian Society of Otorhinolaryngology (SBORL). It was no easy task to make the few Brazilians who published at the time to trust us and send us their papers. In 1996 we had four annual issues, with an average of ten papers per issue and eight advertisers, which amassed profits of R$ 80 thousand per year. Among the main indexing agencies at the time, not only the journal represented a surplus, but it was also the main source of income for the SBORL.

This challenge motivated us to look for the origins and the people - like us, who were also associated with this enterprise and struggled for its survival. In 1990, I published the Memories of the Brazilian Journal of Otorhinolaryngology, in a special issue, and its table of contents and index through the date, then. We managed to collect all the journal issues, since the very first. The special edition is available at the current Brazilian Association of Otorhinolaryngology and Facial and Neck Surgery (ABORL-CCF) and its table of content and index served as a basis to recover all the papers present today in the official website of our journal.

Founded in 1933 with the name of OTO-LARYN-GOLOGICAL JOURNAL OF SÃO PAULO, the publication was headed by Drs. Mário Otoni de Rezende and Homero Cordeiro, who remained at its front until 1963. At that time there was no national body representing the specialty, and most of the specialists in the field practiced Otorhinolaryngology and Ophthalmology. Notwithstanding, the journal published only papers in the field of Otorhinolaryngology, which already hinted at a separation between ENT and Ophthalmology as two independent specialties.

In 1939, the journal became the BRAZILIAN JOURNAL OF OTORHINOLARYNGOLOGY, without disregard for the previous issues, continuing with the numbering started at the Oto-laryngological Journal of São Paulo. In 1964, Dr. José Eugênio Rezende Barbosa became the Journal's Editor, being succeeded by Drs. Rudolf Lang and Nicanor Letti, from 1970 through 1975. Lang remained in that capacity until 1985. At that time, the production was in the hands of the Director of Publications of the Brazilian Society of Otorhinolaryngology, Dr. Arthur Octavio Kós, between 1985-1986, and Dr. Fernando Sérgio Portinho, between 1987 and 1988.

**Graph 1:**
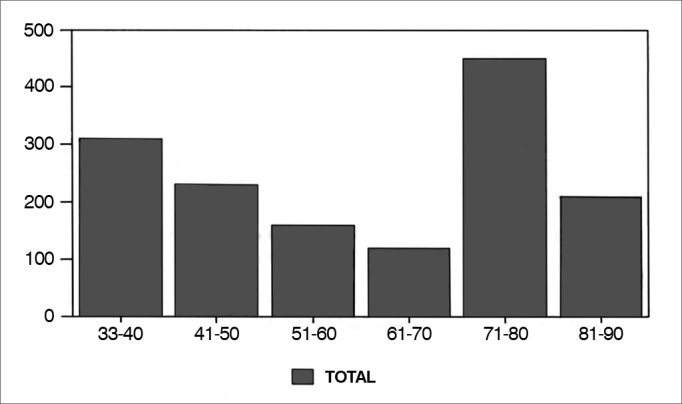
Number of papers on the journal per decade.

In a quantitative assessment of papers, we found 1,461 papers published between 1933 and 1990. In Graph 1 we can appreciate the number of papers per decade. The most productive years of our Journal, from 1930 to 1950 and between 1971 and 1980 had Drs. Mário Ottoni de Rezende and Homero Cordeiro (1930-1950); Rudolf Lang and Nicanor Letti (1971-1980) heading the Journal.

We have to stress our difficulties in publishing scientific papers. Brazilian publications are hammered by international papers, especially those from the United States. In order to correct this shortcoming, in recent years the Journal has been published also in English, aiming at achieving more indexing and exposure, with citations at the ISI. National authors have much difficulty in finding their space in high-impact journals because of prejudice against our publications in developing countries, such as China, Russia and India.

The low government and private stimulus for state-of-the-art scientific research, often times prevents papers from reaching high-impact journals, such as Nature, Science and the New England Journal of Medicine. There is a worsening factor in our specialty: otorhinolaryngology Journals have the lowest impact rates when compared to others. The major publications in our field, the Laryngoscope; Otology and Neurotology; Otolaryngology and Head and Neck Surgery; Rhinology, all have a mean impact rate of 2.0.

University and government research and graduate program agencies, such as CAPES and CNPq, do not respond to the argument that the impact rate in the specific field is most important, and there is a need to match other ENT authors in the world. These committees insist on associating us with other clinical and basic fields which publish in higher impact journals, inherent to their specialties. As the saying goes, they want to “compare apples with oranges”. This is something that has already happened in the USA, Canada and European countries, and in university institutions each field is rated vis-à -vis its international counterparts.

**Graph 2:**
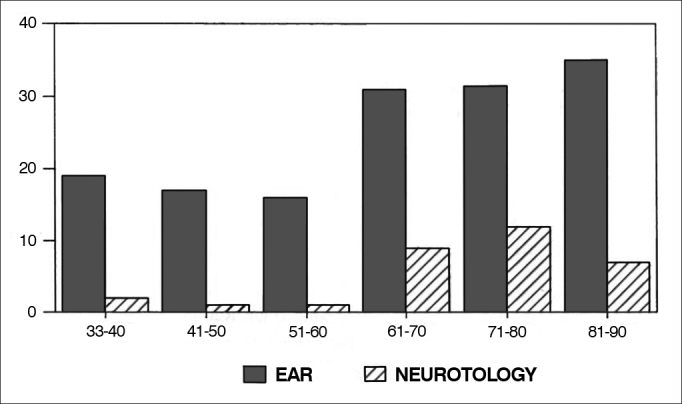
Percentage of ear and neurotology papers in each decade.

Graph 2 shows the percentage of papers on ear and neurotology published in each decade. We can clearly see the evolution of the papers in this field, reflecting the growing interest in this specialty since the 1960's. We can also notice that neurotology alone enjoyed a growing curve between 1960 and 1980, and suffered a drop in 1990.

**Graph 3:**
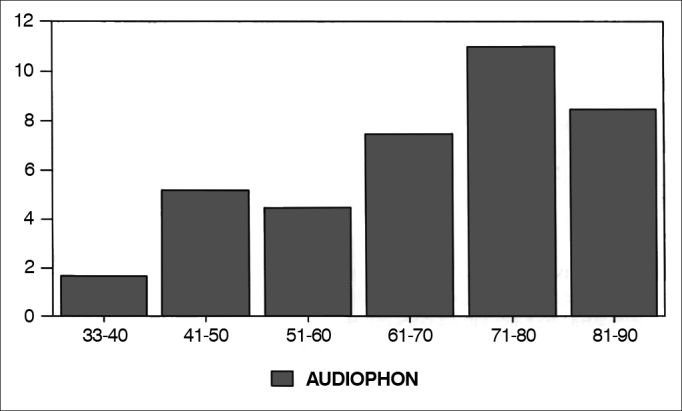
Percentage of audiology and phoniatry papers in each decade.

The so-called Speech-and-Hearing papers, (Graph 3), involving audiology and phoniatry, despite their reduced number, had a growing interest during the 70's and a drop in the 80's.

**Graph 4:**
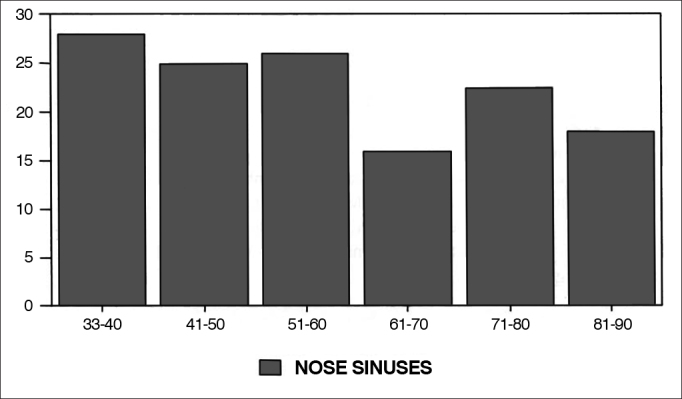
Percentage of nose and paranasal sinuses in each decade.

**Graph 5:**
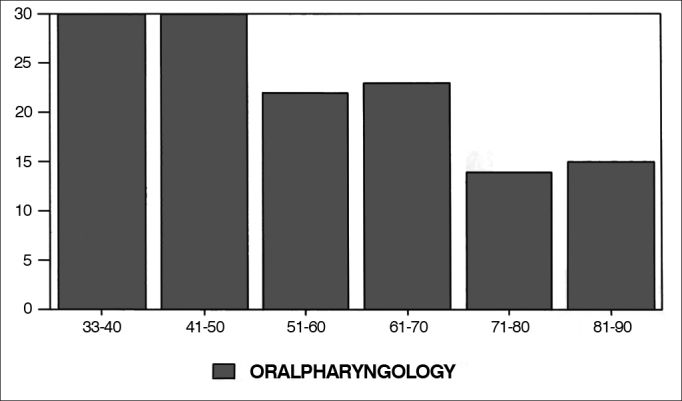
Percentage of oralpharyngology papers in each decade.

**Graph 6:**
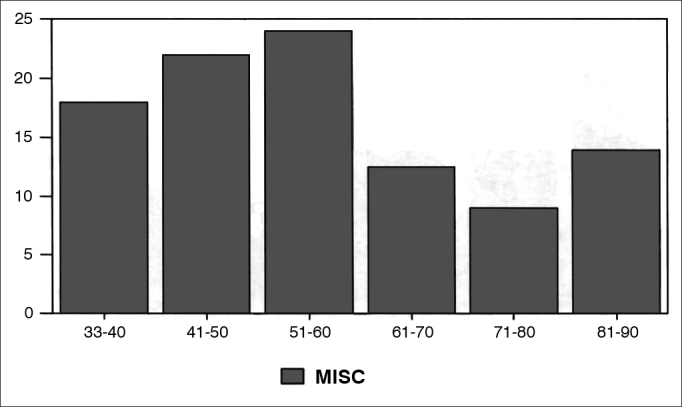
Percentage of miscellaneous papers per decade.

Graphs 4, 5 and 6, dealing with nose and paranasal sinuses, oralpharyngology and head and neck surgery and miscellaneous (papers which do not fit the previous categories) papers had a constant drop between 1930 and 1990, with a mild recovery for nose and paranasal sinuses' papers as of 1970.

Here, I stress some author-related points: During this period, some colleagues had a constant flow of publications, while others published a high number of papers for some time and, then, suddenly stopped, which may coincide with passing in some university program or peaking in their careers. Other colleagues, very productive in terms of attending meetings and conventions, and even famous specialists, are not frequent authors. We could also notice that very productive authors are not necessarily associated with medical schools. Independent specialists or some from private clinics also contribute to our scientific publications.

The goal of these remarks is to stimulate scientific production from lab researchers, academics, graduate students, resident physicians, interns or even independent physicians. Everyone must help with their share, publishing clinical cases, sharing their experience and communicating their techniques. It is only with such procedures that we will grow our field of work.

The Brazilian Journal of Otorhinolaryngology is a true asset of our specialty, and I am proud to be part of its steering team. The work of all the editors who came after me helped increase its credibility and strengthen it among its international counterparts. Today we have the highest possible index mark at ISI, and I continue to believe and invest in the future of our Journal, which success portrays the scientific development of Brazilian Otorhinolaryngology.

